# Successful Treatment of Secondary Aortoenteric Fistula with a Special Graft

**DOI:** 10.1155/2016/9874187

**Published:** 2016-01-03

**Authors:** Ömer Faruk Çiçek, Mustafa Cüneyt Çiçek, Ersin Kadiroğulları, Alper Uzun, Mahmut Ulaş

**Affiliations:** ^1^Department of Cardiovascular Surgery, Türkiye Yüksek Ihtisas Education and Research Hospital, Ankara, Turkey; ^2^Department of Cardiovascular Surgery, Ankara Education and Research Hospital, Ankara, Turkey

## Abstract

Aortoenteric fistula is an uncommon but life-threatening cause of gastrointestinal blood loss. We report a case of a 70-year-old man who presented to the emergency department with an episode of melena and infection in the left inguinal region. Diagnosis of secondary aortoenteric fistula was made between the left limb of the aortobifemoral graft and the descending colon. We performed excision of the infected graft and in situ silver acetate coating of prosthetic vascular graft replacement (aortoleft femoral) on the patient. This study reports a rare type of secondary aortoenteric fistula to the left colon, and it describes an unusual and successful surgical treatment. Antimicrobial coating of prosthetic vascular grafts may be a good alternative in the presence of graft infection associated with aortoenteric fistula because in situ grafts may carry an increased risk of reinfection.

## 1. Introduction

Aortoenteric fistula (AEF) is a rare and life-threatening condition associated with diagnostic challenges. AEFs are classified as primary or secondary depending on their etiology. Primary aortoenteric fistula occurs when an erosive aortic segment opens into the adjacent gastrointestinal lumen. This commonly occurs in abdominal aortic aneurysms. On the other hand, secondary aortoenteric fistula is a complication of aortic reconstructive surgery involving the use of vascular grafts, and it is located between the graft and the adjacent segment of the intestines [1–3].

Secondary AEF has a higher incidence rate than primary AEF, with a reported incidence between 0.36 and 1.6% [3, 4]. On average, 75% of secondary AEFs are located between the distal duodenum and the proximal suture line of aorta, with less frequent involvement of the other gastrointestinal segments. In particular, aortocolonic fistulae are much less frequent, representing 5 to 6% of the cases [5].

In this case report, a very rare form of secondary AEF, an aortocolonic fistula formation, is presented along with a discussion regarding the surgical technique applied.

## 2. Case Presentation

Written informed consent was taken from the patient for publication of this case report. A 70-year-old male patient was admitted to our clinic with abdominal pain and a left inguinal wound with discharge. Approximately 9 months ago, the patient had undergone an aortobifemoral bypass procedure with dacron graft in an another center, after a diagnosis of chronic peripheral arterial disease. In the following course of events, two separate occasions of debridement were performed for the left inguinal lesion with discharge. No history of massive bleeding with hematemesis or hematochezia was present, and the patient was hemodynamically stable at the time of admission. Hematological test results were as follows: Hb = 12.7 g/dL; Htc = 39.3%; WBC = 18.2. Due to the growth of* Staphylococcus aureus* in the wound samples, antibiotic treatment according to the antibiogram results was started. Abdominal ultrasound showed a soft tissue image consistent with infection in the perivascular soft tissue around the left limb of the aortobifemoral bypass graft. Contrast enhanced abdominal computerized tomography (CT) suggested the presence of a suspicious adherence between the left limb of the graft and rectosigmoidal junction. Contrast enhanced CT angiography and digital subtraction angiography (DSA) did not reveal any pathologies suggesting AEF. The patient had diarrhea during his clinical follow-up, and, due to the presence of fresh blood in stool microscopy, a colonoscopy was scheduled by the department of gastroenterology. Colonoscopy showed a blood covered mass lesion with irregular borders located at centrally ulcerative mucosal areas and its appearance was consistent with the graft (see video in Supplementary Material available online at http://dx.doi.org/10.1155/2016/9874187). Since the patient had a prior history of aortic grafting surgery, the findings suggested an AEF and graft infection, which prompted urgent surgical treatment. During the surgery, the left limb of the aortobifemoral graft was observed to be infected and passing transversely into the colon at its distal end. The part of the graft adhering to the colon was separated together with the resection of the same colonic segment, and a colostomy was performed. The left limb of the graft was resected from the bifurcation and from its distal adherence point to the CFA. InterGard Silver polyester graft (Maquet SARL, La Ciotat, France) interposed retroperitoneally into a new tunnel. The proximal part of the silver-coated graft was anastomosed to the resected left limb of the aortobifemoral graft proximally and to the CFA distally. The histopathological examination of the resected colonic segment showed a chronic inflammatory process due to foreign body reaction. Following the operation, the patient completed his course of antibiotics and was subsequently discharged. No postoperative neuromuscular deficits or infections developed postoperatively. Two-year follow-up revealed a complete healing of the inguinal wound and no complaints of claudication.

## 3. Discussion

Secondary aortoenteric fistulae occur as a result of iatrogenic causes, mostly as a complication of aortic interventions. Despite the exact information on its incidence due to a lack of epidemiological data, the estimated incidence is around 1% [6]. Most of the aortoenteric fistulae (75%) occur between the 3rd and the 4th parts of the duodenum and proximal suture line of the aorta, while a small proportion (5%) is located at the colon [5]. In this case report, a rare case of aortoenteric fistula developing between the distal part of the left limb of the aortobifemoral graft and the colon is described.

The pathogenesis of secondary aortoenteric fistulae is controversial with a number of different mechanisms proposed for its occurrence. One of the most significant factors for the development of this condition is the presence of graft infection and inflammation, while another important factor is the presence of a continuous pulsatile movement between the graft and intestines (mechanical factor) [2, 7]. Pseudoaneurysm formed by the dehiscence at the infected suture line is associated with a wear and tear effect in the adjacent intestinal wall, resulting over time in the formation of an aortoenteric fistula. The likelihood of developing a fistula at the suture line between the prosthesis and aorta is higher as compared to the distal suture line [2]. In our case, the fistula formed between the stem of left limb of the aortobifemoral graft and colon, without the involvement of the suture line. This points out to the role of mechanical factors in addition to the infection in the development of the fistula in our patient.

Clinical presentation involves infectious and hemorrhagic components. Most of the symptoms are nonspecific, such as abdominal pain, high fever, bleeding, and shock [2, 7, 8]. The very low incidence of this condition and nonspecific signs and symptoms lead to diagnostic challenges preoperatively, even with the use of advanced imaging modalities. A previous study has found that only 33 to 50% of the cases could be diagnosed appropriately before laparotomy [4]. Contrast enhanced CT is a practical and very useful diagnostic tool. The presence of air or fluid in the perigraft area, inflammation in the adherent intestinal wall, and presence of contrast material in the intestines are highly suggestive of aortoenteric fistula [9]. On the other hand, although colonoscopy can provide useful information, it is generally insufficient for a diagnosis. Colonoscopy is able to detect the location of bleeding in 80% of the cases [10]. Our patient also had nonspecific signs and symptoms at the time of admission such as abdominal pain and discharge from the left inguinal lesion. CT and DSA were not able to detect the passage of contrast medium between the colon and graft, and adhesion between the two structures was suspected. Subsequent colonoscopy and the history of prior aortic surgery allowed us to reach a definitive diagnosis. Examination of the intestinal and graft segments surgically removed showed that the two structures were intermingled. However, due to the absence of complete erosion on the graft wall preventing hemorrhage into the intestinal lumen, the patient did not experience abundant bleeding. Colonoscopy provided very good diagnostic guidance in this specific patient.

Immediately after a diagnosis of secondary aortoenteric fistula is made, the patient has to be operated on as soon as possible, since mortality rate is 100% without treatment [2]. Several surgical approaches are used for the treatment of secondary aortoenteric fistula. But the most widely accepted technique involves the debridement of the infected tissues, repair of the fistula, complete removal of the graft, and revascularization. Axillobifemoral bypass is recommended for maintaining the continuity of distal blood flow [11]. However, the reported risk of amputation for patients undergoing extra-anatomic bypass is 33%, while almost none of the patients who had in situ grafting had extremity loss. This was a main consideration in the decision to preserve the existing graft in our patient. However, recurrent infection risk is high in patients with in situ graft replacement [12]. Imaging studies in our patient showed a weak collateral circulation in the lower extremity. However, since extra-anatomic bypass could result in early extremity loss, in situ replacement with an antimicrobial silver-coated graft was preferred to minimize the risk of infections.

In patients with aortoenteric fistula secondary to previous abdominal aortic surgery, particularly in those with weak collateral circulation of the lower extremity, silver-coated in situ graft replacement may help minimize the early postoperative risk of infections and amputations.

## Supplementary Material

Colonoscopy revealed ulcerative mucosal areas and aortic graft covered by thrombus.
